# Prosimian Primates Show Ratio Dependence in Spontaneous Quantity Discriminations

**DOI:** 10.3389/fpsyg.2012.00550

**Published:** 2012-12-17

**Authors:** Sarah M. Jones, Elizabeth M. Brannon

**Affiliations:** ^1^Department of Psychology and Neuroscience, Duke UniversityDurham, NC, USA; ^2^Department of Psychology, St. Norbert CollegeDe Pere, WI, USA; ^3^Center for Cognitive Neuroscience, Duke UniversityDurham, NC, USA

**Keywords:** non-human primates, numerical cognition, quantity discrimination, prosimians, numerosity, ratio dependence, object file

## Abstract

We directly tested the predictions of the approximate number system (ANS) and the object file system in the spontaneous numerical judgments of prosimian primates. Prior work indicates that when human infants and a few species of non-human animals are given a single-trial choice between two sequentially baited buckets they choose the bucket with the greater amount of food but only when the quantities are small. This pattern of results has been interpreted as evidence that a limited capacity object file system is used to track small numbers of objects and that the ANS is not invoked under these circumstances. Here we tested prosimian primates in food choice comparisons that were chosen to contrast predictions of the ANS and object file systems. We found that prosimian primates consistently chose the larger of two sets when they differed by a 1:3 ratio regardless of whether both values were small (≤3), both values were large (>3), or there was one small and one large value. Prosimians were not able to robustly discriminate quantities that differed by a 1:2 ratio for the same three conditions, nor did they show a preference for small quantities that differed by a 2:3 ratio. These results implicate the ANS in the spontaneous numerical discriminations of non-human primates.

Preverbal human infants and a few non-human animal species have exhibited two contrasting patterns of behavior when faced with quantity judgments. In some tasks, performance is independent of set size and is modulated by the ratio between the two values being compared. In other tasks, successful discrimination is limited to very small values and shows no signs of ratio dependence. This has led to the proposal that there are two cognitive systems that underlie non-verbal numerical discrimination: a limited capacity object file system, which allows the accurate representation of a small number of objects through attentional tracking, and an approximate number system (ANS), which is ratio-dependent and has no upper limit in its capacity (e.g., Uller et al., 1999; Feigenson et al., 2004). The ANS is ubiquitous throughout the animal kingdom and has been shown to operate for large values throughout human development and adulthood (for reviews, see Brannon, 2006; Beran, 2008c). The object file system has been well-documented in human infants under a limited set of circumstances (e.g., Feigenson et al., 2002; Feigenson and Carey, 2003, 2005), and to a much lesser extent, in non-human animals (e.g., Hauser et al., 2000; Agrillo et al., 2007, 2008; Rugani et al., 2008; Uller and Lewis, 2009).

Object files are not explicitly numerical representations, but instead represent individual objects in attention. Each object file “sticks” to a unique object as it moves about the visual scene, and may contain identity or featural information (Kahneman et al., 1992). The object file system represents individuated objects, with the number of open object files providing an implicit way to represent the numerosity of a set. However, as only three or four object files can be maintained simultaneously, the ability of this system to provide a means of representing numerosity is limited to small numbers (e.g., Feigenson et al., 2002; Feigenson and Carey, 2003, 2005; vanMarle, 2013; but see Alvarez and Cavanagh, 2004; Alvarez and Franconeri, 2007).

In contrast, the ANS represents the cardinality of a set of objects as a single mental magnitude. The ability to discriminate between two numerosities in the ANS is ratio-dependent, in accordance with Weber’s Law and is not limited by set size. Small values that are within the capacity of the object file system could be represented with greater precision than the ANS can afford. Thus babies and animals, both of which lack a verbal counting system, could potentially maximize reward in food choice paradigms were they to use the object file system to discriminate small pairs accurately and the ANS to discriminate large pairs approximately.

Studies in animals and human infants typically show ratio dependence across the entire range (e.g., Cantlon and Brannon, 2006b; Beran, 2007; vanMarle and Wynn, 2009) or alternatively show a set size limit such that if either numerosity exceeds the limit, discrimination drops to chance levels of accuracy (e.g., Hauser et al., 2000; Feigenson et al., 2002). However, recent work by Agrillo et al. (2008, 2012) indicate ratio dependence for large values, but not for the values 1–4 in both humans and fish. There is convergent evidence from multiple behavioral paradigms that human infants discriminate between small numerosities (≤3) accurately (e.g., Starkey and Cooper, 1980; Strauss and Curtis, 1981; Wynn, 1992; Koechlin et al., 1997; Feigenson et al., 2002; Feigenson and Carey, 2003, 2005; Xu, 2003; Wood and Spelke, 2005). A subset of these studies have provided strong evidence for the object file system as opposed to the ANS, specifically success with small values (≤3) at a given ratio and simultaneous failure with large sets (>3) at the same ratio (e.g., 2 vs. 3 and 6 vs. 9). The food choice task used by Feigenson and colleagues has repeatedly shown a set size limitation in quantity discriminations in infants (Feigenson et al., 2002; Feigenson and Carey, 2005). In this paradigm, infants are shown food items being dropped into two opaque containers and then allowed to approach one of the containers and consume its contents. Feigenson et al. (2002) demonstrated that 10–12 month old infants reliably crawled to the container with a greater number of food items when both contained three or fewer food items. Thus, 10 and 12 month old infants succeeded at choosing the larger in a 1 vs. 2 and a 2 vs. 3 condition, but performed at chance in a 2 vs. 4 or a 3 vs. 6 condition. Controls for overall duration, complexity, and motivation caused no change to this pattern of performance. In a separate experiment, they demonstrated that infants performed at chance in a 1 vs. 4 condition, but successfully chose the larger in a 0 vs. 4 condition, indicating that infants were capable of representing the existence (vs. non-existence) of crackers, but were unable to compare two numerical values if one exceeded the object file limit (Feigenson and Carey, 2005).

Set size limitations consistent with the object file system have also been reported in numerical discriminations by non-human animals, although there is far more evidence for the ratio-dependent hallmark of the ANS. Hauser et al. (2000) used a single-trial food choice task similar to the food choice task used by Feigenson et al. (2002) with semi free-ranging, untrained rhesus macaques. Monkeys watched as apple slices were placed into each of two opaque boxes. Monkeys were then allowed to approach and consume the apple slices in one box. The monkeys chose the greater number of apple slices as long as the contents of each box did not exceed the set size limit of 4. On comparisons where one box exceeded that limit the monkeys performed at chance, showing no preference for the greater number of food items. This was true even with favorable ratios: 4 vs. 8 and 3 vs. 8. Oddly however, monkeys successfully discriminated 3 vs. 5 in the same study. Wood et al. (2008) demonstrated a set size limitation with non-solid food portions in the same population of rhesus macaques.

Beyond primates, set size limitations have been demonstrated in the spontaneous choices of animals as diverse as horses (Uller and Lewis, 2009), amphibians (Uller et al., 2003), and fish (Agrillo et al., 2007). Agrillo et al. (2007, 2008) found the set size limit (≤3) characteristic of the object file system in the numerical comparisons of mosquito fish, such that fish were more likely to move toward the larger of two shoals in comparisons of 1 vs. 2, 2 vs. 3, and 3 vs. 4, but were not more likely to select the larger shoal for comparisons of 4 vs. 5, 5 vs. 6, 6 vs. 7, or 7 vs. 8. Importantly, Agrillo and colleagues also showed evidence of ratio-dependent performance with large numbers such that they were able to discriminate large numbers at a 1:2 ratio (e.g., 8 vs. 16), but failed at a 2:3 ratio (8 vs. 12).

Two studies have documented a set size limit in the ability to train animals to discriminate between visual arrays. Rugani et al. (2008) trained young chicks to peck at arrays of dots depending on their numerosity. Chicks successfully learned to discriminate 2 vs. 3, but failed to learn to discriminate 4 vs. 6, which suggests that the animals were using the object file system rather than the ANS. Gross et al. (2009) showed a similar result with honeybees: bees successfully learned to distinguish between 2 and 3, but not 4 and 6 items.

One possibility is that untrained animals spontaneously invoke the object file system whenever they are faced with quantity comparisons. However, this hypothesis is not supported by the fact that untrained animals have in some circumstances exhibited ratio-dependent performance indicative of the ANS (e.g., Hauser et al., 2003; Flombaum et al., 2005). Thus, lack of training is insufficient to selectively invoke the object file system over the ANS for numerical comparisons. Nor is the food choice task itself sufficient to reliably tap the object file system. Non-human primates trained and tested on a food choice task have shown ratio-dependent discrimination of simultaneously visible sets (e.g., Anderson et al., 2007; Hanus and Call, 2007; Addessi et al., 2008) and of sequentially presented sets (e.g., vanMarle et al., 2006; Hanus and Call, 2007).

The majority of research on numerical abilities in non-human primates has focused on a few representative species: rhesus macaques, capuchin monkeys, and chimpanzees (e.g., Boysen and Berntson, 1989; Brannon and Terrace, 1998, 2000; Hauser et al., 2000; Cantlon and Brannon, 2006a,b, 2007a,b; Beran, 2007, 2008a,b; Addessi et al., 2008; Beran et al., 2008; Tomonaga, 2008). Very few studies have examined numerical abilities in prosimian primates (e.g., Lewis et al., 2005; Santos et al., 2005; Merritt et al., 2011; Jones et al., submitted). Including prosimian primates in comparisons of primate cognition is likely to be important in attempting to identify cognitive profiles of the primate ancestral state. Prosimian primates have been hypothesized to be morphologically and behaviorally similar to the last common primate ancestor (Tattersall, 1982; Yoder, 2007). Thus, if prosimians primates share cognitive traits that are common among other primates, it is likely that these traits were present in the last common ancestor.

Lewis et al. (2005) showed untrained mongoose lemurs grapes sequentially placed into a bucket with a false bottom. The experimenter surreptitiously hid some subset of the grapes in the false bottom. When the lemurs were allowed to retrieve the grapes from the bucket, they were predicted to search longer if they expected there to be more grapes than they had already retrieved from the bucket. Lewis et al. (2005) reported a pattern of results consistent with ratio-dependent numerical discrimination.

In contrast to the Santos et al. (2005) and Lewis et al. (2005) studies which examined spontaneous numerical discrimination, Merritt et al. (2011) and Jones et al. (submitted) used a touch-screen task to measure numerical comparison abilities in lemurs and macaques. Both studies showed the ratio-dependent hallmark of the ANS in these numerical comparisons. Jones et al. (submitted) tested three different lemur species and macaques and found overlapping numerical acuity for the four species. Thus, to date, both spontaneous numerical comparisons and training have led to evidence for the ANS in lemurs. However, it is important to note that while Lewis et al. (2005) used a spontaneous measure of numerical discrimination, each subject participated in multiple trials. As repeated trials may increase the likelihood of cuing the ANS (vanMarle et al., 2006), it is unclear whether prosimian primates will show a set size limit consistent with object file representations, or the ratio dependence of the ANS, in a single-trial measure of spontaneous numerical comparisons.

Here we use a modified spontaneous food choice task based on Hauser et al. (2000) and Feigenson et al. (2002) to test the spontaneous quantity discriminations of prosimian primates housed at the Duke Lemur Center. We chose a set of numerical values that directly contrasted the predictions of the object file and ANS proposals (see Table 1). We used a 2 × 3 design. There were two numerical ratios (1:2 and 1:3) and three magnitude conditions (small–small, small–large, and large–large).

**Table 1:** **Predictions of the object file system, the ANS, and the two system theory of numerical discrimination for success (√) or failure (x) in each of the conditions of Experiment 1**.

	Quantities	Object file system	ANS	Both systems – incommensurate representations
1:3 ratio	1 vs. 3	√	√	√ (Object file)
	2 vs. 6	X	√	X
	4 vs. 12	X	√	√ (ANS)
1:2 ratio (if 1:2 is beyond the sensitivity limit of the ANS for this task)	1 vs. 2	√	X	√
	3 vs. 6	X	X	X
	6 vs. 12	X	X	X

An object file system would be implicated if: (1) Accuracy was significantly above chance levels of performance only when both numerosities were smaller than the set size limit. (2) Accuracy drops to chance for pairs of numerosities that exceed the set size limit even when the ratio between them is successfully discriminated with smaller numbers (e.g., success at 2 vs. 3 and failure at 4 vs. 6). In contrast, the ANS would be implicated if: (1) Lemurs successfully discriminate pairs with large values, (2) Lemurs show ratio-dependent response functions, with accuracy dropping to chance as the ratio (larger/smaller) approaches 1. A third possibility is that lemurs use object files to represent small values and ANS representations to handle large values but that they are unable to compare incommensurate representations from two different systems and consequently perform at chance on small–large comparisons (e.g., Xu, 2003).

## Experiment 1

### Methods

#### Subjects

Subjects were 113 diurnal and cathemeral prosimian primates (61 females and 52 males; mean age 13.01 years, SD 9.57), housed at the Duke Lemur Center. Each subject participated in one condition with the exception of seven subjects that participated in two conditions, resulting in 120 total trials. Twenty-seven additional trials were excluded due to subject’s failure to participate (*N* = 22) or experimenter error (*N* = 5).

The 120 trials consisted of 20 trials for each of six conditions: two numerical ratios (1:2 and 1:3) and three magnitude pairings (small–small, small–large, and large–large). Participants included individuals from five different genuses and 15 different species (Table 2). Members of each genus were equally distributed among the six conditions, such that each condition contained five *Lemurs*, eight *Eulemurs*, three *Varecia*, three *Propithecus*, and one *Hapalemur*. All animal procedures were conducted in accordance with a Duke University IACUC protocol.

**Table 2:** **A comprehensive list of the species used in each experiment**.

Exp.	Genus	Species	*N*	Sex	Mean age (years)
1	*Eulemur*	*albifrons*	4	2 Females, 2 males	25.64 (SD 3.63)
		*collaris*	5	3 Females, 2 males	23.69 (SD 5.17)
		*coronatus*	6	4 Females, 2 males	15.65 (SD 8.62)
		*fulvus*	1	1 Female	26.64
		*macaco flavifrons*	8	3 Females, 5 males	10.84 (SD 9.91)
		*macaco macaco*	3	1 Females, 2 males	25.17 (SD 3.02)
		*mongoz*	8	4 Females, 4 males	15.31 (SD 8.31)
		*rubriventer*	7	3 Females, 4 males	22.35 (SD 4.04)
		*rufifrons*	1	1 Male	29.03
	*Hapalemur*	*griseus*	5	4 Females, 1 male	14.24 (SD 1.93)
	*Lemur*	*catta*	31	22 Females, 9 males	7.60 (SD 7.89)
	*Propithecus*	*coquereli*	16	8 Females, 8 males	8.65 (SD 7.17)
		*diadema*	1	1 Male	18.26
	*Varecia*	*rubra*	11	4 Females, 7 males	11.18 (SD 9.77)
		*variegate*	6	2 Females, 4 males	11.98 (SD 12.09)
2	*Eulemur*	*collaris*	1	1 Male	18.63
		*coronatus*	1	1 Female	16.87
		*macaco flavifrons*	3	2 Females, 1 male	7.95 (SD 11.21)
		*macaco macaco*	1	1 Male	23.09
		*mongoz*	2	2 Females	13.37 (SD 17.60)
	*Hapalemur*	*griseus*	1	1 Female	13.50
	*Lemur*	*catta*	5	4 Females, 1 male	5.57 (SD 3.80)
	*Propithecus*	*coquereli*	3	1 Female, 2 males	11.54 (SD 5.79)
	*Varecia*	*rubra*	2	1 Female, 1 male	15.51 (SD 16.69)
		*variegate*	1	1 Female	7.00
3	*Eulemur*	*albifrons*	1	1 Female	31.09
		*collaris*	2	2 Females	19.19 (SD 4.16)
		*coronatus*	1	1 Female	24.10
		*macaco flavifrons*	1	1 Male	1.22
		*macaco macaco*	2	1 Female, 1 male	24.71 (SD 3.52)
		*mongoz*	3	2 Females, 1 male	21.30 (SD 5.31)
	*Hapalemur*	*griseus*	1	1 Female	17.24
	*Lemur*	*catta*	2	1 Female, 1 male	5.10 (SD 2.82)
	*Propithecus*	*coquereli*	2	2 Males	9.82 (SD 10.41)

### Procedure

#### Set up

Each subject remained in its home enclosure for testing, but was temporarily restricted from access to cage mates. Each subject was assigned quasi-randomly to one condition. Each trial involved three experimenters. One experimenter operated the camera (E1) and the other two experimenters dropped food items into the buckets (E2 and E3). The numerical conditions were assigned before testing and were known only to E1. E1 gave E2 and E3 each an index card that indicated the number of food items they were to drop into their bucket, which side they were to stand on (left or right), and whether they were to bait the bucket first or second. Experimenters were blind to the number of food items the other experimenter was baiting.

On each trial E2 and E3 stood 2–3 feet apart, immediately outside of the subject’s enclosure, and each held a black bucket that was approximately 30 cm in diameter and 25 cm in height. E1 stood behind the other two experimenters. At the onset of each trial E1 said “start” at which point the two experimenters faced the cage and tipped their buckets on their sides to show the subjects that the buckets were empty.

#### Presentation

E2 and E3 held the buckets with both hands at chest level. E3 closed his/her eyes and remained motionless as E2 baited the bucket with raisins or nuts (depending on dietary restrictions of each species). Each food item was removed from the experimenter’s left breast pocket and held up for the subject to see. Once the experimenter was certain the subject had seen the food item he/she placed it in the bucket. This was repeated until E2 had presented all food items, at which point he/she said “done” and closed his/her eyes. E3 then opened his/her eyes and baited the bucket following the same procedure including stating “done” and closing his or her eyes.

After all food items were presented, E1 determined when the subject had moved to a location approximately equidistant from both buckets and/or averted their gaze from either bucket. E1 then said “buckets down,” at which point E2 and E3 opened their eyes, crouched down and simultaneously set their buckets on the ground against the exterior cage wall. E1 and E2 then stood up, turned 180°, and walked to the other side of the hallway. E1 also turned 180° and watched the subject’s choice via the small finder on the camera.

#### Selection

**Figure 1:**
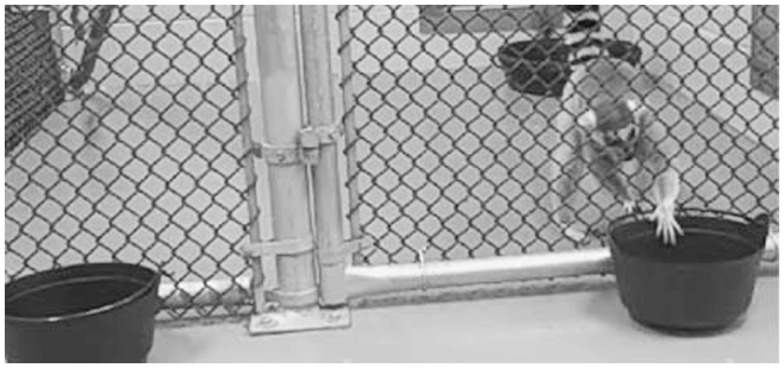
**A photograph of a ring-tailed lemur reaching into one of two buckets**.

A trial ended when E1 determined that the subject had made a choice by moving in front of one of the two buckets and orienting toward it, or when 3 min passed and no choice was made (Figure 1). All data was re-coded by an independent observer who was blind to the hypotheses of the study.

### Results

E1 and the independent observer agreed on which bucket had been chosen on 116 trials (96.67% agreement). For the four trials on which they disagreed, an additional experimenter blind to the condition coded the video and the majority decision was included. Furthermore, the coding of these four trials does not change the reported pattern of results.

**Figure 2:**
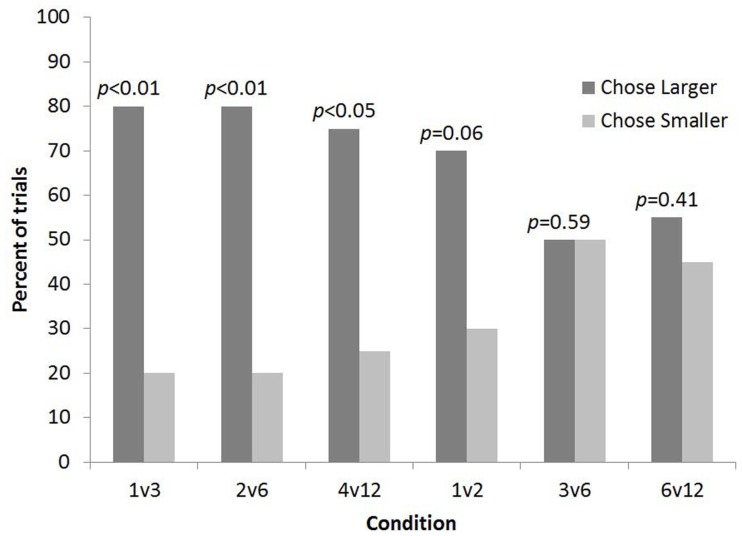
**The percent of trials lemurs chose the bucket with the smaller and larger number of food items for each condition in Experiment 1**.

Overall subjects selected the larger quantity more often than predicted by chance (82 out of 120 trials, *p* < 0.001). Binomial sign tests indicated that subjects chose the larger number of food items significantly more often than predicted by chance for 1 vs. 3 (16 out of 20 trials, *p* < 0.01, one-tailed), 2 vs. 6 (16 out of 20 trials, *p* < 0.01, one-tailed), and 4 vs. 12 (15 out of 20 trials, *p* < 0.05, one-tailed). In contrast, binomial sign tests indicated that subjects chose the larger number of food items no more often than predicted by chance for 1 vs. 2 (14 out of 20 trials, *p* = 0.06, one-tailed), 3 vs. 6 (10 out of 20 trials, *p* = 0.59, one-tailed), or 6 vs. 12 trials (11 out of 20 trials, *p* = 0.41, one-tailed). It should be noted that subjects showed a trend toward selecting the larger number for the 1 vs. 2 condition (*p* = 0.06; Figure 2).

## Experiment 2

Given the trend toward selecting the larger in the 1 vs. 2 condition, the goal of Experiment 2 was to test lemurs with a 2 vs. 3 comparison which comprises a more difficult ratio but should be within the capacity of the object file system.

### Methods

#### Subjects

Subjects were 20 diurnal and cathemeral prosimian primates (13 females and 7 males; mean age 11.16 years, SD 8.74), housed at the Duke Lemur Center. An additional five trials were excluded due to subject’s failure to participate. Subjects represented a similar distribution of species as reported for the conditions in Experiment 1 (Table 2). Due to a limited number of naïve animals available for testing, 4 out of the 20 subjects had already participated in Experiment 1.

### Procedure

The procedure was identical to Experiment 1.

### Results

**Figure 3:**
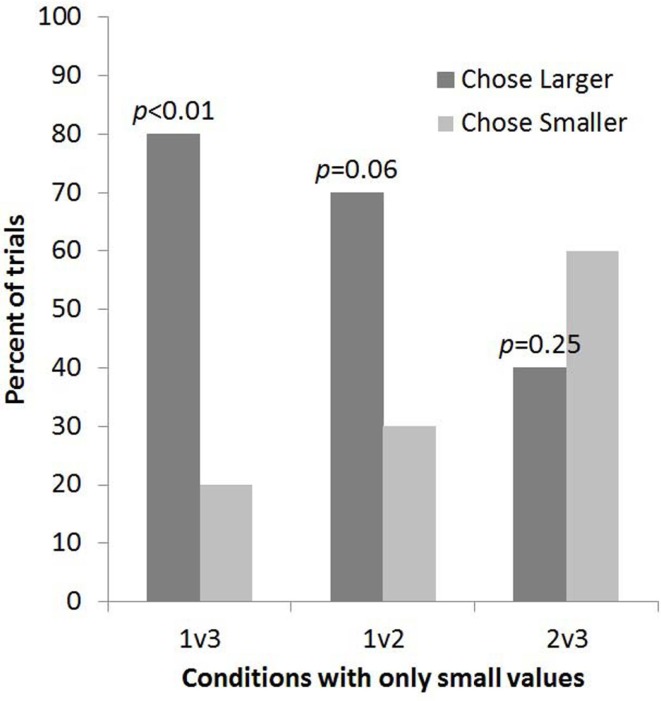
**The percent of trials lemurs chose the bucket with the smaller and larger number of food items for each condition in Experiment 2 (2 vs. 3 condition) alongside data from the other small number comparisons tested in Experiment 1**.

Subjects selected the larger quantity no more often than predicted by chance for 2 vs. 3 (8 out of 20 trials, *p* = 0.25, one-tailed; Figure 3). The four subjects who had been tested in Experiment 1 showed no consistent pattern of responding and the exclusion of these trials would not change the pattern of results: one chose the larger in both experiments, one chose the smaller in both experiments, one chose the larger in Experiment 1 but the smaller of 2 vs. 3, and one chose the smaller in Experiment 1 but the larger of 2 vs. 3.

## Experiment 3

The goal of Experiment 3 was to provide a scent control. To this end we tested lemurs with a 2 vs. 6 comparison and pre-baited the bucket that was designated for the smaller quantity such that the two buckets provided the same olfactory cues.

### Methods

#### Subjects

Subjects were 15 diurnal and cathemeral prosimians (eight females and seven males; mean age 17.01 years, SD 9.53), housed at the Duke Lemur Center. Subjects represented a similar distribution of species as in Experiment 1 (Table 2). Thirteen out of 15 subjects had been previously tested in Experiment 1.

### Procedure

The procedure was identical to Experiment 1 except that all trials consisted of a 2 vs. 6 comparison in which the bucket that was baited with two already contained four food items hidden in the bottom. This meant that when the baiting was complete, both buckets contained six food items providing the same olfactory cues^1^.

### Results

Subjects chose the bucket into which they had observed six food items placed significantly more often than predicted by chance (12 out of 15 trials, *p* < 0.05, one-tailed).

## General Discussion

Our findings provide little support for the idea that prosimian primates use object file representations to track food items. Instead the data are consistent with the idea that lemurs spontaneously represent and compare quantities using the ANS. Subjects were able to successfully select the larger quantity with a 1:3 ratio but not a 1:2 ratio or a 2:3 ratio. The fact that lemurs were able to successfully discriminate two from six is also counter to the predictions of incommensurate representations. Failure to differentiate two from three food items further suggests that ratio dependence rather than set size limited their performance. It is important to note that this does not indicate that lemurs are incapable of discriminating small values. Indeed, our results indicate that they are just as capable of discriminating small values as large values. Instead, this points to a ratio-dependent system that is equally sensitive across magnitudes.

Is the reason our results differ from others due to genus or species differences? Other work from our research group suggests that lemurs and monkeys have quantitatively similar numerical discrimination capacities (Jones et al., submitted). In that study, rhesus macaques, ring-tailed lemurs, mongoose lemurs, and blue-eyed black lemurs were trained to select the numerically larger of two visual arrays on a touch-screen. Despite the large variation in social structure, home range size, and diet in the species tested, all four species showed similar weber fractions. Thus, we find it unlikely that the lack of evidence for a set size limit in the spontaneous numerical comparisons of lemurs reflects a difference between prosimian primates and old world primates. Alternatively, the lack of evidence for a set size limit reported here may reflect subtle differences in the testing conditions in our study and the prior studies with rhesus macaques (Hauser et al., 2000; Wood et al., 2008). Candidates for these factors include, but are not limited to, satiation, inhibition, arousal, competition, dominance, and inadvertent social cues from the experimenter. For example, testing conditions differed from Hauser et al. (2000) in that while the macaques were semi-free-ranging, the subjects in the present study were caged and were separated briefly from conspecifics during testing to reduce interruptions and competition. Future research will need to address the contexts that cue the object file system in spontaneous discrimination tasks.

While we did include Experiment 3 to provide a scent control condition, we did not include a control for auditory cues or total duration. Previous research using this task have included such controls with infants and monkeys and resulted in no change to the pattern of responding (Hauser et al., 2000; Feigenson et al., 2002). Given these previous findings, we believe it is unlikely that controls for auditory cues or duration would impact performance in this task however we cannot rule out these alternative possibilities.

We made an additional modification of the protocol used by Hauser et al. (2000) that may account for different patterns of results by attempting to eliminate the possibility of a *Clever Hans effect*. In the majority of quantity discrimination research, the experimenters presenting food items have been aware of which container held the larger quantity. It is thus possible that subjects made selections based on unintentional social cues from the experimenters. We established a simple modification to the design, which allowed the experimenters presenting food items to be blind to the condition on any given trial. At the time of testing, each experimenter was given an index card that indicated the number of food items they were to drop into their bucket and they were unaware of the number being baited in the other bucket.

A number of authors have proposed that small quantities may be represented by both ANS and object file systems, and that contextual factors may determine which system is cued (e.g., Wynn et al., 2002; Feigenson, 2005; Barner et al., 2008; Cordes and Brannon, 2008, 2009; Hyde, 2011). The simplest possibility is that different systems are used when animals make spontaneous judgments compared to when they perform tasks for which they have extensive training. Hauser and others suggested that the object file system might be primary when animals engage in spontaneous numerical judgments without training and that extensive training might be required for animals to represent large values outside the purview of the object file system (Hauser et al., 2000; vanMarle et al., 2006). However, we tested untrained animals in the same spontaneous cognition circumstances and found no evidence for the object file system. These results emphasize the importance of selecting values that can directly contrast the predictions of two systems and test the limits of each system.

Others have proposed more nuanced explanations for the contextual factors that elicit object file vs. ANS representations. For example, a recent study showed that exact enumeration of small numbers (<4) is inhibited during a task with high attentional load, but approximate numerical representation is not (Burr et al., 2010; but see Vetter et al., 2008, for contrasting evidence that the enumeration of both small and large numbers is equally affected by attentional resources). Another study showed that individual differences in small number representation correlated with working memory, but ANS acuity did not (Piazza et al., 2011). Hyde and Wood (2011) suggested that spatial attention impacts which system will represent the numerical value of a small set (1–3 items). Specifically, they report that when the spatial distribution of visual objects allowed for individuation, ERP responses showed a pattern consistent with parallel individuation. In contrast, when attention could not select individual objects, ERP responses showed a pattern consist with ratio dependence. Hyde (2011) hypothesized that conditions that allow attentional selection of individuals cue the object file system, while conditions in which items are presented outside attentional limits result in approximate numerical representations. Our findings do not support this hypothesis: small quantities were presented sequentially without additional attentional requirements, and yet still resulted in approximate representations.

Other explanations for the contextual factors that elicit object file vs. ANS representations involve features of the sets, such as heterogeneity and movement. Feigenson and colleagues (Feigenson et al., 2002; Feigenson, 2005) reported a double dissociation in infants’ representations of small object arrays such that infants responded to changes in the numerosity of heterogeneous, but not homogeneous arrays when area is controlled for. Additionally, several authors have proposed that the movement of items within a set may impact which system of representation is elicited. Wynn et al. (2002) and Barner et al. (2008) suggested that objects which undergo common motion are more likely to be represented as a collective entity than objects that move independently. For example, when all objects within a set move together, two sets of five elements may be more likely to be perceived as two entities than as 10 independent objects. Thus, common motion may result in an array being represented as a single set with an approximate numerical magnitude.

As our paradigm involved individually presented food items, we cannot address these hypotheses regarding heterogeneity or common motion. It is clear, however, that multiple contextual factors appear to be involved in eliciting object file or approximate representations, rather than a simple explanation in which different systems are used for spontaneous judgments and trained numerical tasks. It is important to note that we do not claim that our results mean that lemurs, or non-human primates more generally, never use the object file system. Rather, we argue that a spontaneous food choice task is not a sufficient context to elicit a set size limit on quantity discrimination and that the ANS is robust even over these spontaneous decisions.

In sum, by employing conditions designed to specifically address the predictions of the ANS and the object file system, we found that spontaneous numerical comparisons in prosimian primates are likely to be driven by the ANS. Given these results, the factors that may lead non-human primates to compare quantities using the object file system rather than the ANS remain unclear. What is clear, however, is that the ANS is spontaneously accessed by non-human primates to compare quantities regardless of the magnitude of those values being tested.

## Conflict of Interest Statement

The authors declare that the research was conducted in the absence of any commercial or financial relationships that could be construed as a potential conflict of interest.

## Footnotes

^1^In contrast to Experiments 1 and 2, experimenters were not blind to the number of food items the other experimenter was baiting.
